# Hydroxyapatite-Based Colloidal Gels Facilitate the Proliferation and Migration of Chondrocytes and the Adhesion of Umbilical Cord Mesenchymal Stem Cells

**DOI:** 10.1155/2014/935689

**Published:** 2014-12-29

**Authors:** Syed A. Jamal, Qiang Ye

**Affiliations:** ^1^Division of Molecular Biosciences, University of Kansas, Haworth Hall, 1200 Sunnyside Avenue, Lawrence, KS 66045, USA; ^2^Rockhurst University, Kansas City, MO 64109, USA; ^3^Ascend Technology, Dallas, TX 75034, USA; ^4^School of Engineering, University of Kansas, Lawrence, KS 66045, USA

## Abstract

Collective movement of cells that have been delivered on biomaterials for transplantation purposes would be a desirable attribute that would promote wound healing, cell proliferation, and eventual growth and regeneration of damaged organs. We hypothesized that colloidal gels made from hydroxyapatite (HA) and poly(D,L-lactic-co-glycolic acid) (PLGA) particles will be conducive to the growth and migration of porcine chondrocytes, will allow the adhesion of human umbilical cord mesenchymal stem cells, and will have negligible effects on the cell cycle of these cells. Then, we performed experiments designed to assess the viability and migratory properties of porcine chondrocytes studded on nanosized HA/PLGA particles. Our experiments show that porcine chondrocytes migrated in and around a hydroxyapatite-based biomaterial that could be described as a colloidal gel. Cells in the colloidal gel demonstrated unidirectional movement. Cells were seen to be extending lamellae and were followed by other cells.

## 1. Introduction

Hydroxyapatite (HA), Ca_10_(PO_4_)_6_(OH)_2_, which resembles bone in composition and biocompatibility, has been widely used in tissue engineering approaches aimed at regeneration of bone and teeth [1]. PLGA happens to be a biodegradable material that has been extensively used in pharmaceuticals and in tissue engineering scaffolds [2]. Scaffolds made from PLGA nanoparticles have been shown to exhibit negligible toxicity and optimal rheological properties [3–6]. Injection of cells loaded on biocompatible materials such as hydroxyapatite that can withstand the shear force exerted by normal wear and tear of the human body and that can degrade in a timely, predictable manner can help in achieving regeneration, especially in cases of skull injuries where intramembranous ossification can lead to direct differentiation of mesenchymal cells into bone-forming osteoblasts [7]. Once cells are seeded on a biomaterial, coordinated cell movement is a quintessential requirement for cellular proliferation, differentiation, and subsequent tissue regeneration [8]. Cell movement is influenced by chemical gradients, substrates, extracellular tension, and electrostatic potential [8]. Cartilage is composed of mature chondrocytes surrounded by a matrix: these chondrocytes are usually dormant; however, chondrocytes are active, during fetal life when cartilage matrix is soft [9]. Chondrocytes can use their actomyosin machinery to generate the force needed to detach themselves from the matrix and propel themselves forward; presumably, such a scenario would require proteolytic breakdown and subsequent rebuilding of the matrix machinery—a process that has been observed in fetal articular cartilage in lambs [9, 10].

In vivo motility of chondrocytes remains an unexplored area [9]. In this study, we employed three-dimensional colloidal gels made of HA/PLGA/chitosan to study the viability and migration of porcine chondrocytes and found that porcine chondrocytes could move in culture around hydroxyapatite. Our results indicate that colloidal gels made from hydroxyapatite aid and abet the migration of cells in culture and have a salutary effect on the cell cycle.

## 2. Materials and Method

### 2.1. Gel Formation

Cell culture studies were carried out on gels provided by Cory Berkland's Lab at the University of Kansas. For atomic force microscopy (AFM) and scanning electron microscopy/energy dispersive X-ray analysis (SEM/EDAX) experiments, HA nanoparticles were a gift from Dr. Carole McArthur's Laboratory at the University of Missouri and School of Medicine and Dentistry at the University of Kansas while PLGA was synthesized using a modified version of the method used by Khodaverdi et al. [11]. Lactic and glycolic acid were mixed in equal proportions in a 150 mL beaker; trace amounts of 700 *μ*M mercury and stannous chloride were added to the beaker; the beaker was heated at high power in a microwave for 3 cycles of 4 minutes each. A viscous product was obtained that was dried in an oven at 180°C. The hydroxyapatite nanoparticles were dissolved in this PLGA, sterilized in UV light overnight, and characterized by SEM. Chitosan was obtained from shrimp shells using a procedure modified from that used by Hadi [12]. Demineralized shrimp shells were soaked in 4% Hcl overnight and rinsed profusely with water to remove acid and calcium chloride. For protein removal, the demineralized shells were treated with 5% NaOH (12 : 1 solvent to solid ratio) and heated at high power in microwave for 1 hour. The residues were collected and washed to neutrality with distilled water. Dried residues were subjected to heat for 1 hour at 180°C to achieve deacetylation [11–13].

### 2.2. Cell Culture

Human umbilical cord mesenchymal stem cells (hUMSCs) obtained from human placenta were a gift from Dr. Michael Detamore's Laboratory. Porcine chondrocytes were harvested from the ankles of pigs as per the protocols approved by the Committee on Ethics (IRRB) at the University of Kansas. Cells were grown in low glucose Dulbecco's Modified Eagle's Medium, 10% fetal bovine serum (FBS), and penicillin/streptomycin. Then, chondrocytes were seeded at a density of 1000 cells per cm^2^ and grown to confluence in a 12-well tissue culture-treated plate.

Cells were seeded on colloidal gels, made from hydroxyapatite and PLGA/chitosan, for up to two to six weeks. Next, viability assays were conducted. MTS assay: MTS assay on porcine chondrocytes seeded on HA/PLGA biomaterials: MTS assay was performed according to the manufacturer's protocol (Promega). MTS assay was utilized to assess cell viability. Cells in culture for 15 days were used for the assay. 100 *μ*l of cells in culture media containing 50000 cells was seeded in culture media on colloidal gels made from 30/70, 70/30, and 50/50 HA/PLGA/chitosan that were used for cell viability assay. 20 *μ*l of Cell Titer 96 AQ (Promega) MTS reagent was added to the wells. After incubation at 37°C for 3 hours, the plates were read at 490 nm.

### 2.3. Characterization of Composition and Surface Morphology of the Biomaterial by Raman Spectroscopy and AFM

#### 2.3.1. Raman Spectroscopy

Raman spectra of HA/PLGA/chitosan, HA/PLGA/chitosan with cell culture media, and HA/PLGA/chitosan with cells were obtained with a 100x objective using a He/Ne laser with a 60-second acquisition time, in the 100–3200 cm^−1^ range (a Horiba Jobin Yvon Labram ARAMIS fully automated confocal Raman imaging system was used).

AFM, operating in tapping mode, was used to characterize the surface properties of the colloidal gels (Multimode V Atomic Force Microscope from Veeco Instruments, Santa Barbara, California).

The sample was made by pouring the colloidal gel inside a silicone tube. An oscillating cantilever tip at its resonance frequency was allowed to come into contact with the surface of the material and then scanned over the material; as the oscillation of the cantilever is affected by the surface topography, the feedback controller maintains the amplitude of the cantilever at a fixed set point value. The vertical movements of the cantilever tip needed to keep a constant amplitude yield the topography map; the phase map comes from the delay in oscillation of the tip that occurs in response to the excitation force [14].

### 2.4. SEM and EDAX

A Versa 3D dual beam Scanning Electron Microscope/Focused Ion Beam (FEI, Hillsboro, OR, USA) with a silicon drift EDX detector (Oxford Instruments, X-Max, UK) was used to measure the surface morphology, elemental composition, and distribution of elements. All the SEM data reported were obtained at an acceleration voltage of 10 kV, spot size 4.0, and the images were collected with an ET (Everhart Thornley) detector.

### 2.5. Microslide Experiments

30 *μ*l of cell suspension with 40000 cells (porcine chondrocytes) was injected to fill the microslide chamber. 30 *μ*l of media only was placed on the other side of the chamber. Next, biomaterial was placed in between the two chambers. As our experiment turned out, there was no need to use a chemoattractant.

### 2.6. Real-Time Imaging and Cell Tracking

We used a phase contrast microscope (TE2000; Nikon Instech, Tokyo, Japan) equipped with a 10x objective (numerical aperture 0.3) for time-lapse observations. A series of time-lapse images were captured every 5 min using a high-resolution digital charge-coupled device camera controlled by Image-Pro software (Media Cybernetics, Silver Spring, MD). Observations were started 24 h after plating the cell suspension on the Petri dish. A movie was edited from the series of captured images. A link to the movie is available in the Supplementary Material (available online at http://dx.doi.org/10.1155/2014/935689). The web url is https://www.youtube.com/watch?v=1o4aECP3fu4.

### 2.7. DRAQ Staining of Porcine Chondrocytes

DRAQ5 stock solution was diluted in PBS. 100 *μ*l of this solution was added to each well of the tissue culture flask containing live chondrocytes. The cells were then excited with laser light of 562 nm, viewed under 40x, and emission was captured in the far red.

### 2.8. Statistical Analysis

Student's *t*-test was performed on Microsoft Excel with a significance determination at *P* < 0.05. Results are shown as mean ± standard error.

## 3. Results and Discussions

### 3.1. Viability Studies

We observed very little death in cells treated with HA/PLGA/chitosan nanoparticles for 48 h (Figure 7). On the other hand, appreciably higher numbers of cells died when 70% ethanol was used as a negative control (unpublished data).

Our live/dead experiments have previously shown that HA/PLGA colloidal gels have little cytotoxicity towards hUMSCs, suggesting that the material may be used as a scaffold for seeding chondrocytes as well [15, 16]. In this experiment, the results of our MTT assay and the spectra from Raman spectroscopy demonstrate that our HA/PLGA/chitosan biomaterial supports the growth of chondrocytes as well.

### 3.2. Cell Attachment as Viewed by Time-Lapse Video Microscopy

**Figure 1:**
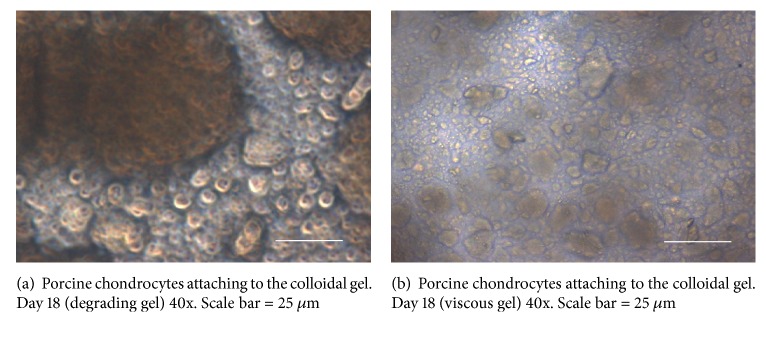
Bright field microscopy of porcine chondrocytes cultured for 18 days on colloidal gels. In (a), the biomaterial gel has broken down. In (b), the cells are in between the colloidal gel and some cells are sitting atop the gel.

As seen in the micrograph in Figure 1(a), chondrocytes are seen on top of the biomaterial, which is mostly compact in areas where bright efflorescence is observed (Figure 1(b)). Darker areas represent the biomaterial without enough cellular attachment.

### 3.3. Degradation of the Biomaterial

Our colloidal gel biomaterial had degraded into a fluid cluster phase by around 3 weeks as seen in Figure 1(b). This is an interacting system of colloid particles that interact with each other via van der Waals forces: the gel breaks up due to the exertion of shear forces by cellular movement into aggregates of particles floating in the culture medium.

### 3.4. Biomaterial Characterization

#### 3.4.1. Surface Morphology of the Biomaterial by AFM

The surface of the biomaterial, as shown in Figure 3(a), would provide a smooth surface punctuated by rough areas or ridges—that cells would latch onto without sliding—and by pores to facilitate passage of nutrients.  Figure 3(b) clearly indicates a smooth surface. A biomaterial with rough surface containing grooves and ridges promotes the attachment and movement of cells [17].

#### 3.4.2. Scanning Electron Microscopy and Composition by Energy Dispersive X-Ray Analysis (EDAX)

The image in Figure 5(b) clearly shows nanosized particles with pore sizes that would allow passage of nutrients.

#### 3.4.3. Raman Spectroscopy

**Figure 2:**
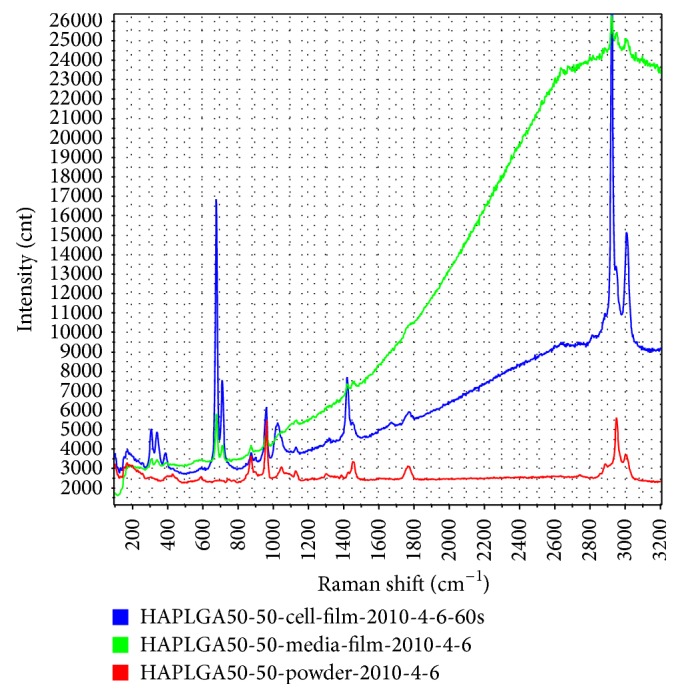
Raman spectrum obtained from porcine chondrocytes cultured on HA/PLGA gels. Red spectrum is from HA/PLGA only; green spectrum comes from HA/PLGA gel plus media; and blue spectrum is derived from HA/PLGA/chitosan gels that had cells cultured on them.

**Figure 3:**
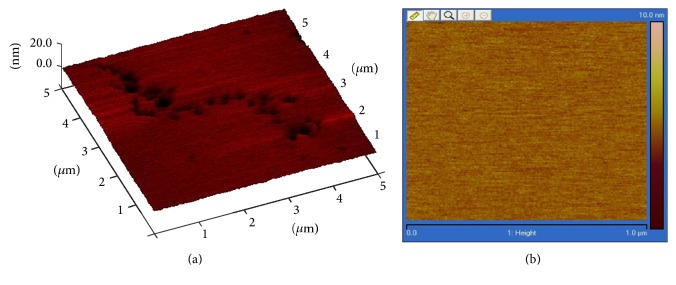
AFM micrograph obtained from the 30–70 HA/PLGA/chitosan colloidal gels. (a) In the 5 μm by 5 μm scan size, raised, darker regions represent ridges and nanosized pores. (b) The control shown here, as a 1 μm by 1 μm scan of the image shown in 3(a), displays a smooth, flat region that is thoroughly consistent.

As seen in the Raman spectrum in Figure 2, the HA/PLGA/chitosan biomaterial gave a strong peak at 960 cm^−1^ and weak signals at 450 and 600 cm^−1^; these signals were indicative of hydroxyapatite [18–20]. As seen in the green peak, there was some noise beyond 1800 cm^−1^ from the biomaterial in cell culture media. In the spectra with cells, peaks were obtained for cytoplasmic proteins at 690, adenine at 729, phenylalanine at about 1005, and methylene scissor vibration from lipid chain in plasma membrane [18–20].

**Figure 4:**
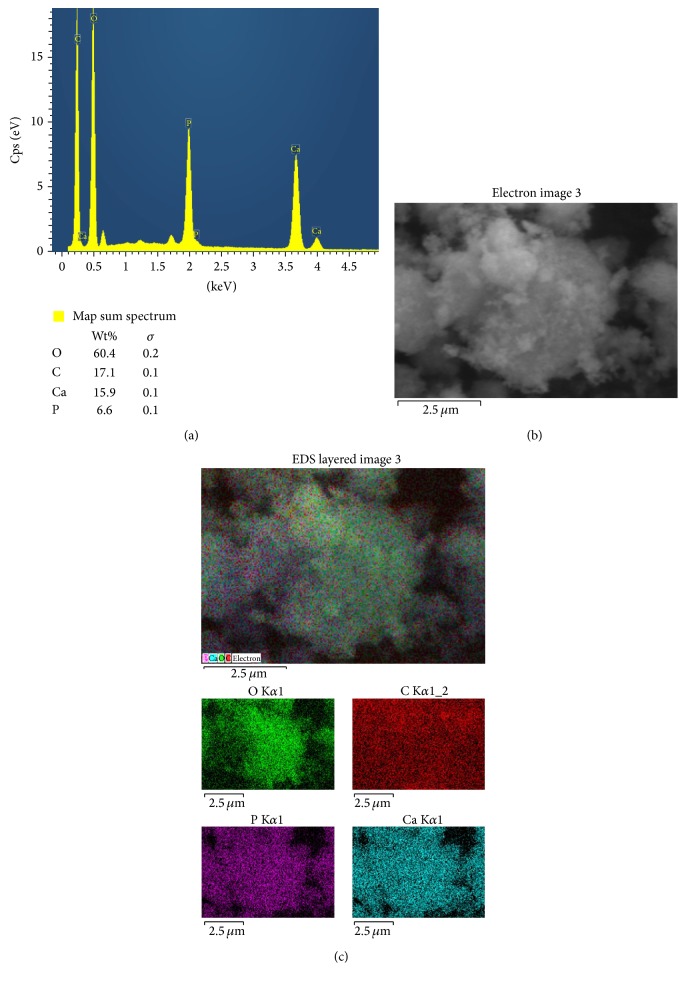
(a), (b), and (c) EDAX analysis of HA/PLGA/chitosan biomaterial.

*EDAX Analysis*. As shown in Figures 4(a), 4(b), and 4(c), HA/PLGA/chitosan shows the presence of Ca, P, O, and C atoms. The SEM micrograph in Figure 4(b) clearly indicates the amorphous nature of PLGA in the biomaterial. Interestingly, the dimensions of the pores are within the nanometer range capable of providing large surface area for enhanced cell attachment and exchange of materials. In Figure 4(c) top panel displays a collage of the images obtained from the separate layers denoting the elements present as shown in the colored EDS spectra in the bottom of Figure 4(c).

### 3.5. Cell Migration and Movement

**Figure 5:**
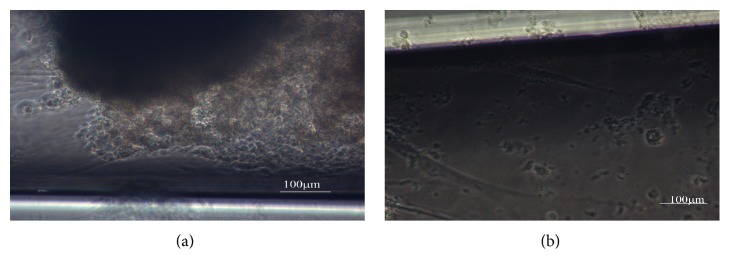


As shown in Figures 5(a) and 5(b), round porcine chondrocytes surround the biomaterial around 2 days after seeding. There is little matrix between the cells as would be expected in fibrous cartilage surrounding ankle joints [20]. These chondrocytes cross the zone separating the 2 wells within 4 days.

It is interesting that these cells can move around and through the biomaterial as our live imaging studies have shown (link to youtube: https://www.youtube.com/watch?v=1o4aECP3fu4).

### 3.6. DRAQ5 Staining of Nuclei

**Figure 6:**
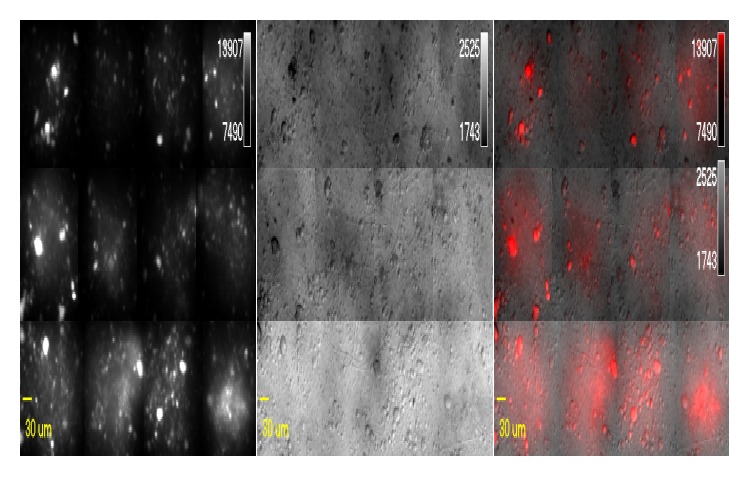
DRAQ staining of chondrocytes demonstrating healthy nuclei. Some cells with bright intensity are those cells expected to be in the S phase of the cell cycle as evidenced by intense staining of the oval nuclei.

Staining experiment showed that chondrocytes had viable nuclei. As seen in Figure 6, some nuclei stained much more intensely than others: these are quite likely the mitotically dividing chondrocytes and the dense stain is given by clump of cells in the S phase since chondrocytes are small, oval cells, about 10–30 *μ*m in diameter suspended in a homogeneous extracellular matrix [12]. Since DRAQ5 dye is only known to stain the nuclei of living cells, viable nuclei can be observed as white dots.

Our results clearly show that the combination of HA, PLGA, and chitosan can yield a nontoxic biomaterial that can be developed into an injectable colloidal gel supporting cell attachment as well as cell migration needed for the purposes of regenerative pharmacology and tissue engineering.

**Figure 7:**
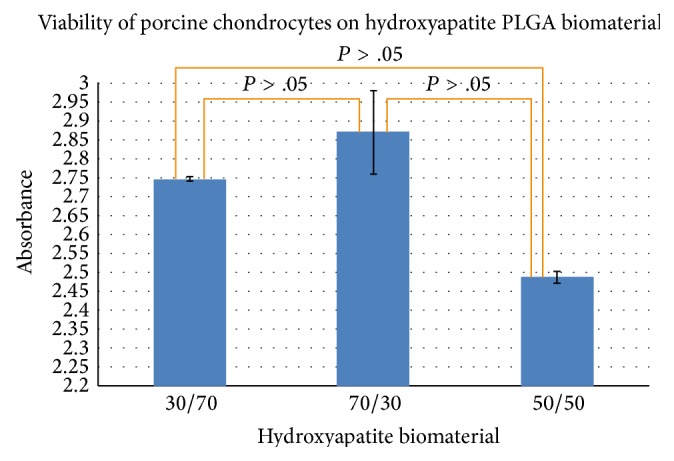
MTS assay. Porcine chondrocytes were viable on 30/70, 70/30, and 50/50 colloidal gels made from hydroxyapatite/PLGA/chitosan. Cell growth was similar on all three different compositions of the biomaterial.

Our colloidal gels showed desirable porosity, though pore size needs further optimization. Long-term live cell experiments allowed us the luxury of visualizing cell migration on IBID chamber and observing interactions between cell membrane adhesive proteins and the components of our colloidal gel [8]. Cooperative interactions between the forces of elasticity, gravity, and surface tension induce surface folding in the gel that leads to attraction and motion of particles in the gel. Cell movement, as determined by the time it took cells to cross over into opposite chamber as the cells moved over and through the biomaterial, was slower than that observed in our real-time imaging studies. Since our cells were embedded in the biomaterial, it is, therefore, highly likely that the elastic nature of our gel played a role in the movement of cells. On day 4, porcine chondrocytes had crossed the zone into the other chamber of the microslide. The amount of degradation observed confirms the possibility of optimizing degradation kinetics of the gel to facilitate cell growth. Another tantalizing possibility that we can discern from our experiments is that collective movement of chondrocytes may, together with the release of proteases, be a factor in the breakdown of the extracellular matrix, in the same manner in which collective movement of the chondrocytes likely helped degrade the biomaterial in our study. While mature chondrocytes are enshrouded in a proteoglycan rich pericellular matrix and a basket-like territorial matrix filled with fibrillar collagen, immature chondrocytes might contain chondroprogenitor cells as well as a matrix that is progressively being laid down [9]. The colloidal gels were found to be cytocompatible (Figure 7), and an appreciable degree of cell adhesion was seen in light microscopy (Figure 1).

## 4. Conclusions

To our knowledge, this is the first study of collective movement of porcine chondrocytes in and around colloidal gel-like biomaterial. While elasticity studies have been conducted on HA and on PLGA, the stiffness of the biomaterial that we used has not been well characterized; viscosity studies have been difficult because the colloidal gel tended to get fractured; and, importantly, visualization of cell nuclei using fluorescent dye on the biomaterial was hindered due to interference by hydroxyapatite, especially blurred image formation due to scattering [15]. Lest our study is viewed as merely phenomenological, we stress that our experiments were carefully designed to facilitate the movement of cells and to capture these movements as well. Instead of focusing on individual cell movement, we were more interested in following collective cell movement that involves interacting cells and the breakdown of matrix material. We have shown that cells residing within colloidal gels can move, and this proof of concept study can be taken further by delivering cells in gels to sites in animal model studies, especially for cartilage development. One of the shortcomings of our study resides in the fact that no phenotypic analysis of chondrocytes was undertaken; another weakness stems from the fact that biomarkers typical of chondrocytes such as GAGs (glycosaminoglycans) or alcian blue staining characterizing sulfated proteoglycans of functional chondrocytes were not screened.

Our rationale was that the chondrocytes were isolated from porcine ankles and viewed under microscope. As regards the functionality of these chondrocytes, our video clips show cells moving, and only functional cells display motility. Future studies using gels of varying stiffness should address to what extent the softness or the relative stiffness of our biomaterial facilitated cell mobility as seen in our experiments and elucidate the understanding of cell migration astride colloidal gels.

## Supplementary Material

Supplementary material information is
